# Characteristics of motor evoked potentials in patients with peripheral vascular disease

**DOI:** 10.1371/journal.pone.0290491

**Published:** 2024-04-25

**Authors:** Pawandeep Sarai, Charlotte Luff, Cyrus Rohani-Shukla, Paul H. Strutton

**Affiliations:** The Nick Davey Laboratory, Division of Medicine, Department of Surgery and Cancer, Imperial College London, London, United Kingdom; Harvard Medical School, UNITED STATES

## Abstract

With an aging population, it is common to encounter people diagnosed with peripheral vascular disease (PVD). Some will undergo surgeries during which the spinal cord may be compromised and intraoperative neuromonitoring with motor evoked potentials (MEPs) is employed to help mitigate paralysis. No data exist on characteristics of MEPs in older, PVD patients, which would be valuable for patients undergoing spinal cord at-risk surgery or participating in neurophysiological research. Transcranial magnetic stimulation, which can be delivered to the awake patient, was used to stimulate the motor cortex of 20 patients (mean (±SD)) age 63.2yrs (±11.5) with confirmed PVD, every 10 minutes for one hour with MEPs recorded from selected upper and lower limb muscles. Data were compared to that from 20 healthy volunteers recruited for a protocol development study (28yrs (±7.6)). MEPs did not differ between patient’s symptomatic and asymptomatic legs. MEP amplitudes were not different for a given muscle between patients and healthy participants. Except for vastus lateralis, disease severity did not correlate with MEP amplitude. There were no differences over time in the coefficient of variation of MEP amplitude at each time point for any muscle in patients or in healthy participants. Although latencies of MEPs were not different between patients and healthy participants for a given muscle, they were longer in older participants. The results obtained suggest PVD alone does not impact MEPs; there were no differences between more symptomatic and less symptomatic legs. Further, in general, disease severity did not corelate with MEP characteristics. With an aging population, more patients with PVD and cardiovascular risk factors will be participating in neurophysiological studies or undergoing surgery where spinal cord integrity is monitored. Our data show that MEPs from these patients can be easily evoked and interpreted.

## Introduction

Motor evoked potentials (MEPs) are used extensively as a research tool to explore neuromuscular function in health and disease [1–3]. Changes in characteristics of MEPs have been observed in many neurological and neuromuscular pathologies, such as stroke and muscular dystrophies [3, 4] and for intra-operative neuromonitoring (IONM) to diagnose and mitigate spinal cord injury during spinal surgery or thoraco-abdominal aortic aneurysm (TAAA) repair [5, 6].

Patients undergoing spinal surgery and TAAA repair are often older and frequently have cardiovascular risk factors such as high blood pressure, hypercholesterolaemia and smoke [7, 8]. As such, they frequently have concomitant peripheral vascular disease (PVD) in addition to their presenting pathology, where the arteries of the lower limbs are atherosclerotic, leading to occlusion and subsequent chronic limb ischaemia (CLI) [9]. This in turn leads to tissue loss and damage of the limb nerves and muscles.

MEP characteristics have been exhaustively studied in heathy volunteers, and in specific spinal or neurological disease [10–13]. The MEPs of PVD patients, however, have not previously been examined. Understanding changes that occur due to CLI is not only beneficial for IONM, but for any research or clinical situation where MEPs need to be characterised in patients with cardiovascular risk factors or recognised PVD. With an aging population and increasing rates of obesity and cardiovascular disease [14], the co-incidence of PVD will inevitably increase.

Current standards of IONM utilise MEPs generated by transcranial electrical stimulation (TES) [15]. However, TES in the awake participant can be prohibitively painful. Transcranial magnetic stimulation (TMS) is an alternative technique for generating MEPs, with the benefit of being pain-free, [16] thus it is frequently employed in neurophysiology research [17]. Further, MEP changes seen pre- and intra-operatively with TMS, correlate with TES-induced MEP changes during surgery and clinically after surgery [17, 18]. TMS is therefore suitable to characterise MEPs in non-anaesthetised PVD patients in the research and surgical settings.

The aim of this study was to determine the characteristics of TMS-induced MEPs from limb muscles in PVD patients and compare these to data from participants without cardiovascular risk, or PVD, which formed part of a protocol development study.

To our knowledge, this not been previously studied and would provide essential baseline data for future neurophysiological study of patients with cardiovascular disease and risk factors, with possible clinical applications, including intraoperative and post-operative monitoring.

## Material & methods

### Participants

20 patients with PVD (17 males), mean (±SD) age of 63.2 (±11.5) years took part in the study. All patients were symptomatic with radiologically confirmed PVD under the care of a vascular surgical team. Data were also collected prior to this study from 20 participants (8 females; 28 (±7.6) years of age) with no co-morbidities or cardiovascular risk factors. Recruitment occurred between 15^th^ November 2016 and 20^th^ November 2018.

A TMS safety questionnaire was undertaken to ensure appropriateness for inclusion [19]. The inclusion and additional PVD-specific exclusion criteria for patients are listed in Table 1.

**Table 1:** Patient-specific inclusion and exclusion criteria.

Inclusion Criteria	Exclusion Criteria
Experience symptomatic lower limb claudication pain (cramping in lower leg when walking), not currently suitable for surgery	Age < 18yrs
Under regular review by the Vascular Surgery Department at Imperial College NHS Trust for peripheral vascular disease (the build-up of fatty deposits in the arteries (blood vessels) that restrict blood supply in the lower legs) and not currently suitable for surgery.	Diabetes Mellitus (High blood sugar level) with no know microvascular complications e.g. peripheral neuropathy
	Confirmed or possibility of pregnancy
	Open wounds or skin lesions, including wet gangrene (dry gangrene permitted)
	Inability to speak and understand English fluently
	Patients with known thoracic or thoraco-abdominal aneurysm awaiting repair

This study was approved by the Imperial College London research ethics committee (number: 16IC3553) and National Health Service health regulatory authority (number: 17/LO/1034) and performed with adherence to the Declaration of Helsinki guidance on conducting research in human participants. Written informed consent was obtained from all participants.

### Disease severity questionnaire (VascuQol)

Patients completed the King’s College Hospital’s Vascular Quality of Life Questionnaire (VascuQol); this validated questionnaire determines disease severity with 25 questions exploring the effects of PVD on physical and emotional well-being of patients [20]. Each question is graded from 0 to 7, with 0 indicating PVD affects a particular aspect of their life all of the time (i.e. no ability to perform task). A mean score of 4.5 or less is associated with symptomatic intermittent claudication due to CLI [21].

### Electromyography

**Fig 1:**
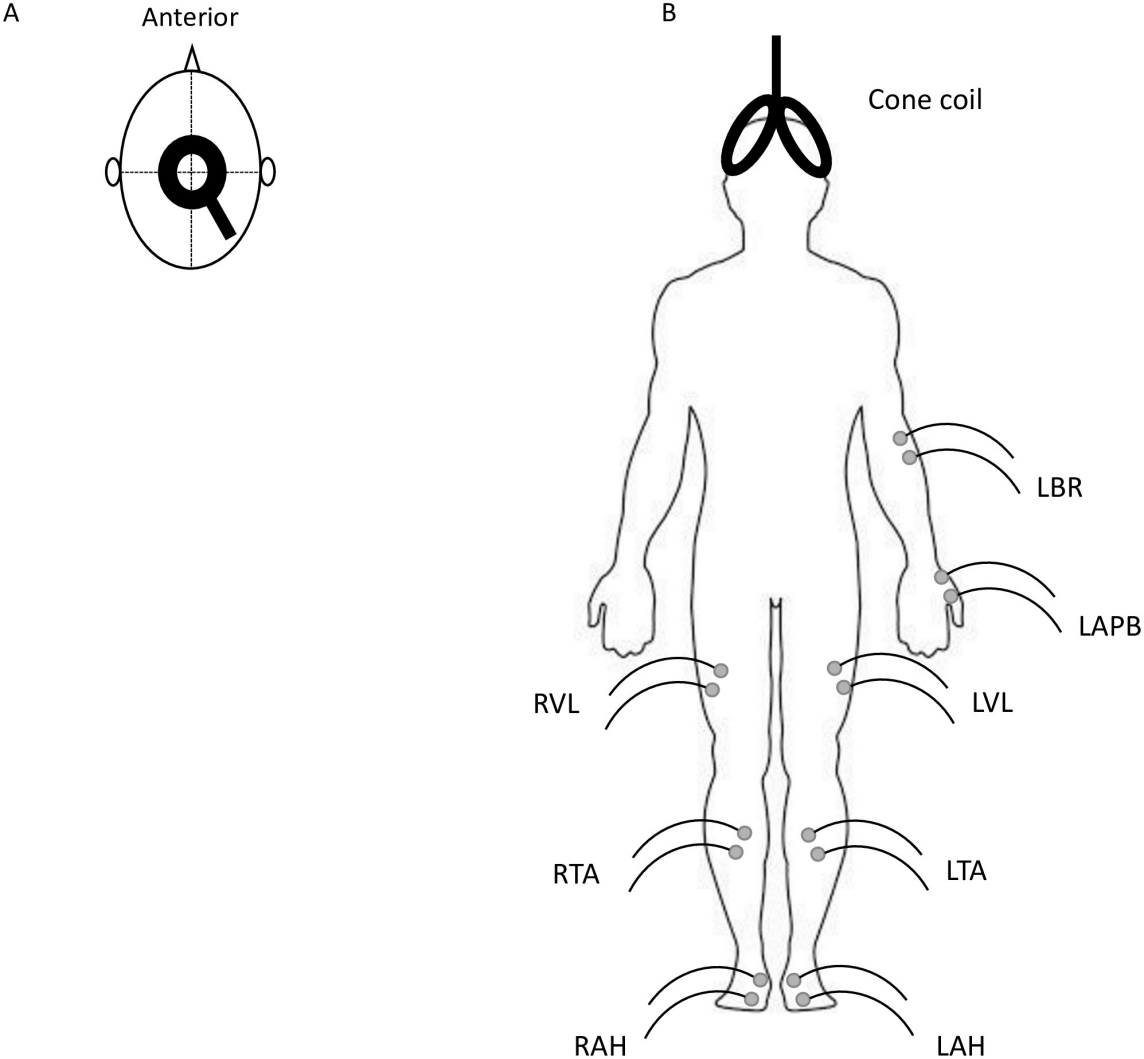
A) Illustration of circular coil placement over vertex, B) Electrode placement on PVD patients with cone coil shown for illustration. Ground electrode over olecranon process omitted. L = Left, R = Right, BR = brachioradialis, APB = abductor pollicis brevis, VL = vastus lateralis, TA = tibialis anterior and AH = abductor hallucis.

With participants supine, pairs of disposable Ag/AgCl electrodes (25mm diameter, 1041PTS, Henleys Medical Supplies Ltd., UK) were applied to the skin 20mm apart, overlying the muscles of interest, after skin preparation with alcohol [22]. To explore potential differences in disease severity between the left and right leg, patients had electrodes placed over left and right vastus lateralis (VL), tibialis anterior (TA) and abductor hallucis (AH), as well as left brachioradialis (BR) and left abductor pollicis brevis (APB), see Fig 1. A ground electrode was placed over the ulna olecranon process. These limb muscles were chosen as they are supplied by different major peripheral nerves.

Raw EMG signals were amplified (x1000), band-pass filtered (10Hz–1kHz) (ISO Dam, WPI, UK) and sampled (at 2kHz) using an analogue to digital converter (Power 1401, Cambridge Electronic Design [CED], UK). Signal software (CED, UK) was used to record to disk for subsequent analysis.

### Transcranial magnetic stimulation

TMS was delivered using two Magstim 200^2^ magnetic stimulators (Magstim, Dyfed, UK), each connected to a different coil, which was positioned over the vertex. The location of the vertex was determined as the point on the scalp at which two lines, from nasion to inion and from tragus to tragus, bisect; this point was marked with an indelible pen to ensure consistent coil placement. A circular coil (90mm outer diameter), which is more commonly used to stimulate many upper limb muscles simultaneously, was positioned B-side up to induce anticlockwise current in the brain, thus preferentially activating the right hemisphere [23]. An angled double-cone coil (wing outer diameter 120mm), which is capable of stimulating deep into the motor cortex due to its construction and geometry, was employed to stimulate lower limb muscles. It was positioned over the vertex so that the induced current in the brain flowed in the posterior-to-anterior direction. Two ‘combined’ resting motor thresholds (cRMT) were determined for the upper and lower limb muscles for each participant, with the circular and cone coils, respectively. This was the lowest stimulation intensity that produced MEPs ≥50μV in amplitude [24] in at least three out of six stimuli in either all upper or lower limb muscles. Stimulator intensities of 120% of the cRMT for the muscle with the highest threshold were used [25]; these are shown in Table 2 for both study groups. For a given coil, there were no differences between intensities used between healthy participants and patients; circular coil P = 0.62, cone coil P = 0.19.

**Table 2:** Stimulation intensities (mean maximum stimulator output (%MSO) and range) for upper and lower limb muscles for healthy volunteers and patients.

	Upper limb muscle stimulation intensity (%MSO)	Lower limb muscle stimulation intensity (%MSO)
Healthy participants	56 (42–69)	55 (44–70)
Patients	55 (44–68)	58 (43–71)

Each block of stimulation consisted of six single stimuli from each coil, every 10 minutes over the course of 60 minutes (seven sets of twelve stimuli in total from 0 to 60 minutes). Participants were instructed to relax all muscles during stimulation to prevent facilitation [26].

### Data analysis

#### Patient disease severity questionnaire (VascuQol)

The scores from 25 questions were averaged to give a mean value from 0 to 7. Microsoft Excel 2016 (Microsoft, USA) was used to perform calculations.

#### Motor evoked potentials

Signal Software (CED, UK) was used for data analysis by visual positioning of cursors at the start and finish of each MEP. Peak-to-peak amplitude and latency (time from stimulation to first MEP deflection) were subsequently measured.

Given the known variability of TMS-induced MEPs in relaxed muscles [27] the variability both within a time point and across the whole experiment was calculated. The variability between measurements was determined by the coefficient of variation (CV); CV = standard deviation/mean.

*Variability within a time point*. To determine the variability at a given time point, the amplitude of each MEP was measured, and the CV of the 6 MEPs calculated. The CV of the MEP amplitude was calculated for each time point and for each muscle.

*Variability across the whole experiment*. The averaged MEP (avMEP) is a digital averaging process, where the 6 MEPs at a given time point are amalgamated to produce a single MEP; from this the amplitude and latency are measured.

To determine the variability of MEPs across the duration of the experiment, the amplitude of the avMEP at each of the 10-minute time points was measured and the overall CV calculated. This was performed for each muscle and each participant to calculate the mean CV of the avMEP amplitude; comparisons over time for a given muscle were performed.

### Statistical analysis

Statistical analyses were carried out using GraphPad Prism v10.1.2 (GraphPad Software LLC, Boston, MA, USA). Data were tested for normality (Shapiro-Wilk) and appropriate tests (non-parametric) were performed with post-hoc multiple comparisons with Dunn’s corrected P values reported.

AvMEP amplitudes, CVs of the avMEP amplitudes, AvMEP latencies and CVs of the avMEP latencies were collapsed over the hour and compared between patient-reported symptomatic and asymptomatic sides in patients. Where both legs were symptomatic, data were evenly split between groups to allow equal group sizes for analysis. No differences were found between symptomatic and asymptomatic legs with respect to these parameters, hence data were averaged between legs and compared between patients and healthy participants using Kruskal-Wallis tests.

To examine changes in variability of MEP amplitudes within a time point over the hour protocol, CV of MEP amplitudes were compared over time and between symptomatic and asymptomatic lower limb muscles using Kruskal-Wallis tests. Since no differences were found between symptomatic and asymptomatic legs, data were averaged between legs. To determine relationships between clinical score (VascuQol) and MEP characteristics in patients and between age and MEP characteristics across all participants, and between avMEP amplitudes and CV of avMEP amplitudes, Spearman’s correlations were performed.

The level of statistical significance was set at P<0.05, or appropriate for multiple comparisons. Data are presented as mean ± SD in the text and figures.

## Results

### VascuQol score in PVD group

The VascuQol score was 4.3±1.2, representing symptomatic PVD not requiring surgical intervention (as per the main inclusion criteria). This score indicates patients experience cramp-like pain in the lower leg when walking due to muscle ischaemia.

### Amplitude of avMEP

**Fig 2:**
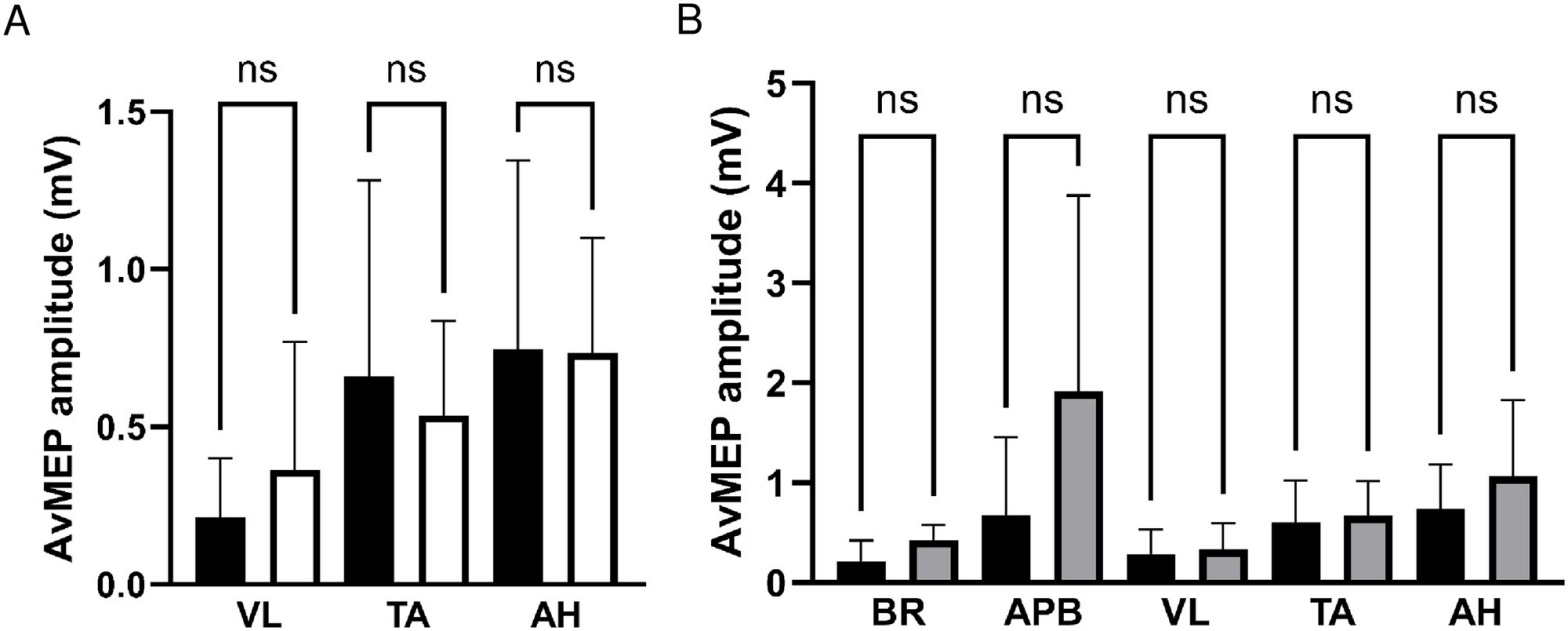
AvMEP amplitude (mean ± SD) for A) patient lower limb muscles VL, TA and AH comparing symptomatic (black bars) and asymptomatic (white bars) legs, and B) upper limb muscles BR and AHB and lower limb muscles VL, TA and AH comparing patients (black bars) and healthy participants (grey bars). NS represents no significant difference.

There were no differences in avMEP amplitudes between symptomatic and asymptomatic legs in patients (VL, 0.21±0.19mV vs 0.36±0.41mV, P>0.99; TA, 0.66±0.62mV vs 0.54±0.30mV, P>0.99; AH, 0.75±0.60mV vs 0.73±0.37mV, P>0.99), see Fig 2A; data were therefore averaged between sides.

There were no differences between avMEP amplitudes of patients and healthy participants for a given muscle (Fig 2B).

### CV of avMEP amplitude

**Fig 3:**
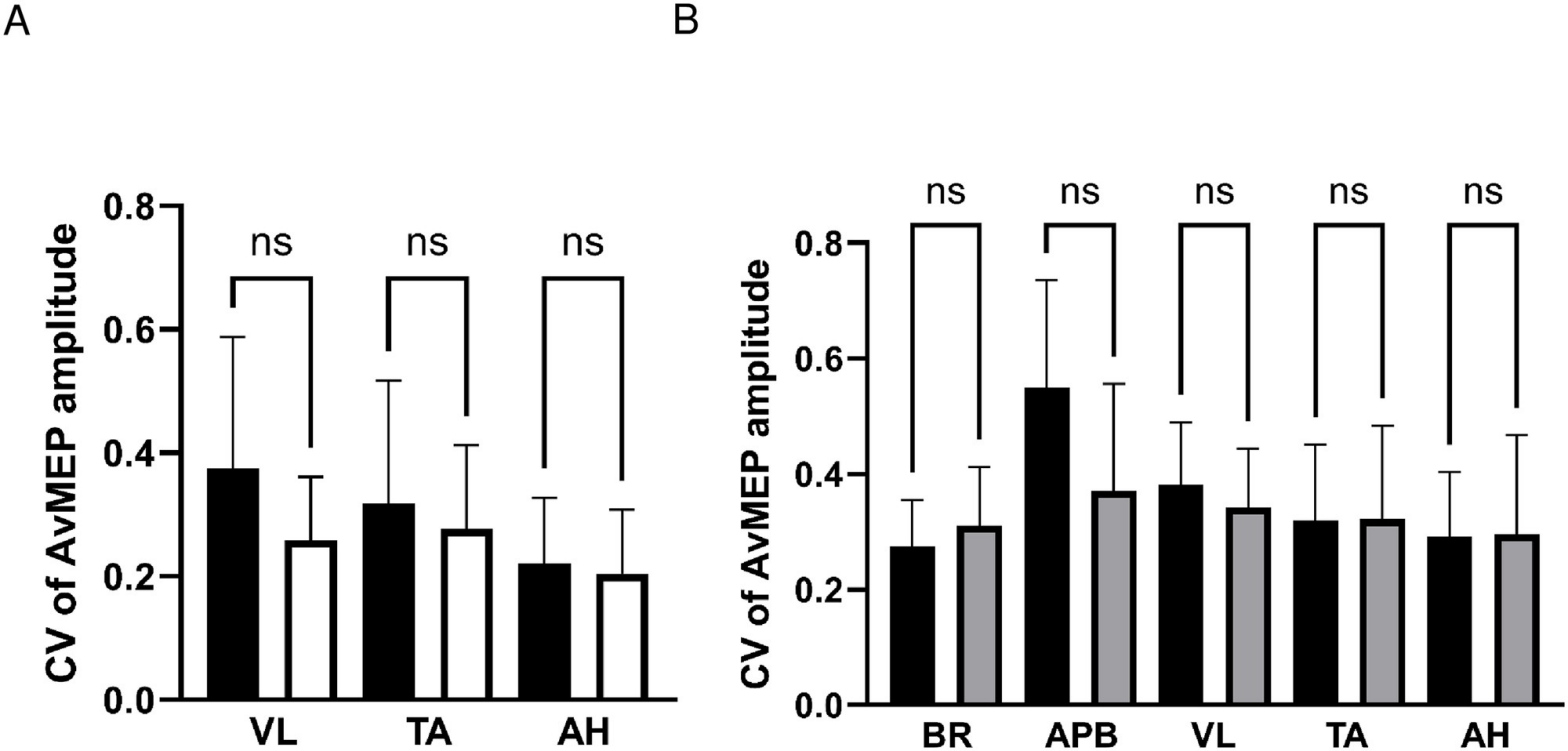
CV of AvMEP amplitude (mean ± SD) for A) patient lower limb muscles VL, TA and AH comparing symptomatic (black bars) and asymptomatic (white bars) legs, and B) upper limb muscles BR and AHB and lower limb muscles VL, TA and AH comparing patients (black bars) and healthy participants (grey bars). NS represents no significant difference.

There were no differences in CV of avMEP amplitudes between symptomatic and asymptomatic legs in patients (VL, 0.38±0.21 vs 0.26±0.10, P>0.99; TA, 0.32±0.20 vs 0.28±0.14, P>0.99; AH, 0.22±0.11 vs 0.20±0.10, P>0.99), see Fig 3A; data were therefore averaged between sides.

There were no differences between CV of avMEP amplitudes of patients and healthy participants for a given muscle (Fig 3B).

### Latency of avMEP

**Fig 4:**
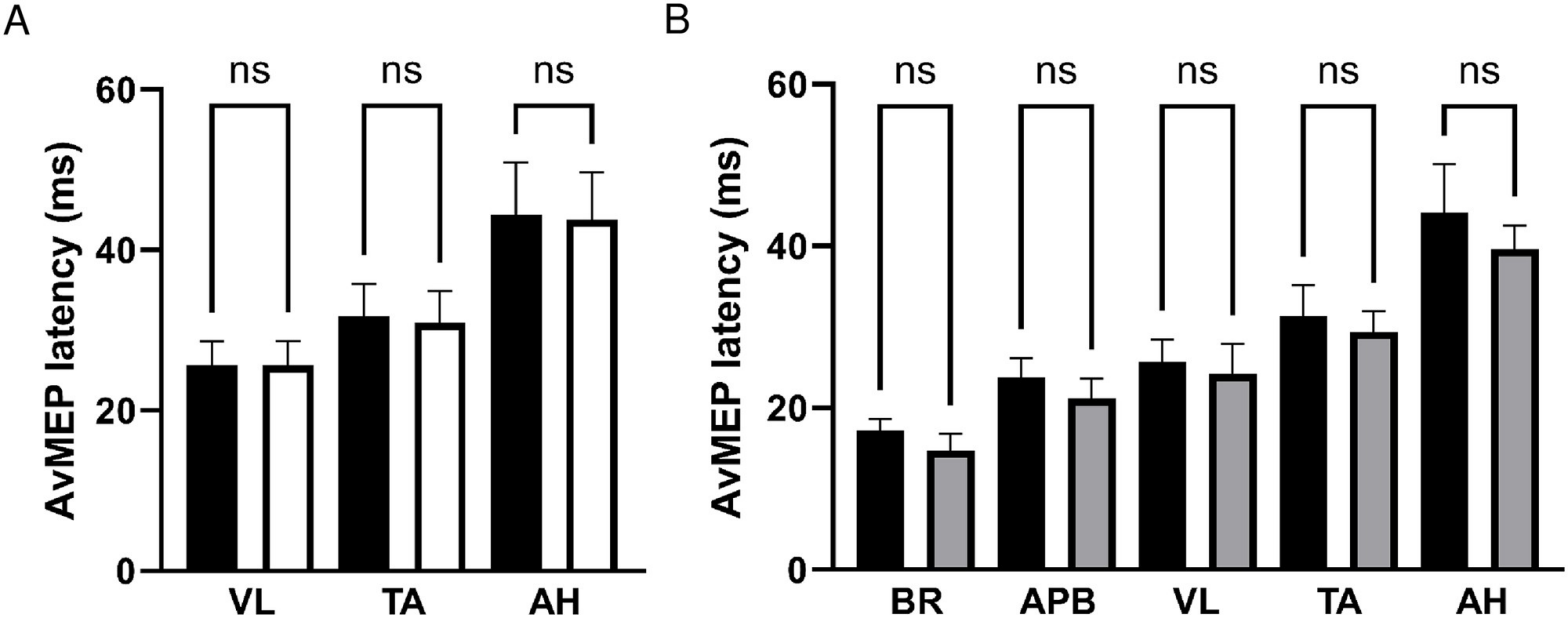
AvMEP latency (mean ± SD) for A) patient lower limb muscles VL, TA and AH comparing symptomatic (black bars) and asymptomatic (white bars) legs, and B) upper limb muscles BR and AHB and lower limb muscles VL, TA and AH comparing patients (black bars) and healthy participants (grey bars). NS represents no significant difference.

There were no differences in avMEP latencies between symptomatic and asymptomatic legs in patients (VL, 25.62±3.00ms vs 25.63±3.05ms, P>0.99; TA, 31.74±4.04ms vs 30.93±3.94ms, P>0.99; AH, 44.37±6.50ms vs 43.78±5.88ms, P>0.99), see Fig 4A; data were therefore averaged between sides.

There were no differences between avMEP latencies of patients and healthy participants for a given muscle (Fig 4B).

### CV of avMEP latency

**Fig 5:**
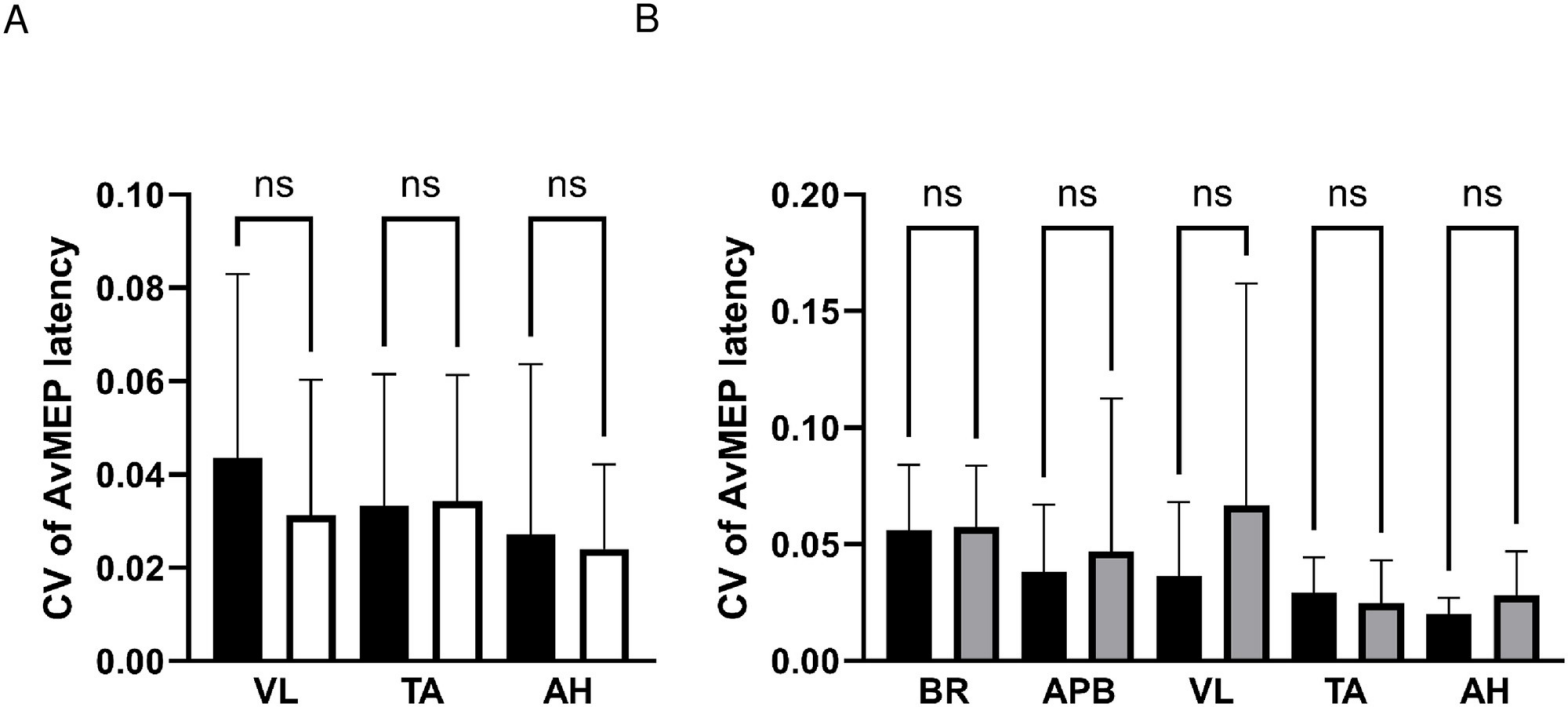
CV of AvMEP latency (mean ± SD) for A) patient lower limb muscles VL, TA and AH comparing symptomatic (black bars) and asymptomatic (white bars) legs, and B) upper limb muscles BR and AHB and lower limb muscles VL, TA and AH comparing patients (black bars) and healthy participants (grey bars). NS represents no significant difference.

There were no differences in CV of avMEP latency between symptomatic and asymptomatic legs in patients (VL, 0.04±0.04 vs 0.03±0.03, P>0.99; TA, 0.03±0.03 vs 0.03±0.03, P>0.99; AH, 0.03±0.04 vs 0.02±0.02, P>0.99), see Fig 5A; data were therefore averaged between sides.

There were no differences between CV of avMEP amplitudes of patients and healthy participants for a given muscle (Fig 5B).

### Mean CV of MEP amplitude at each time point

**Fig 6:**
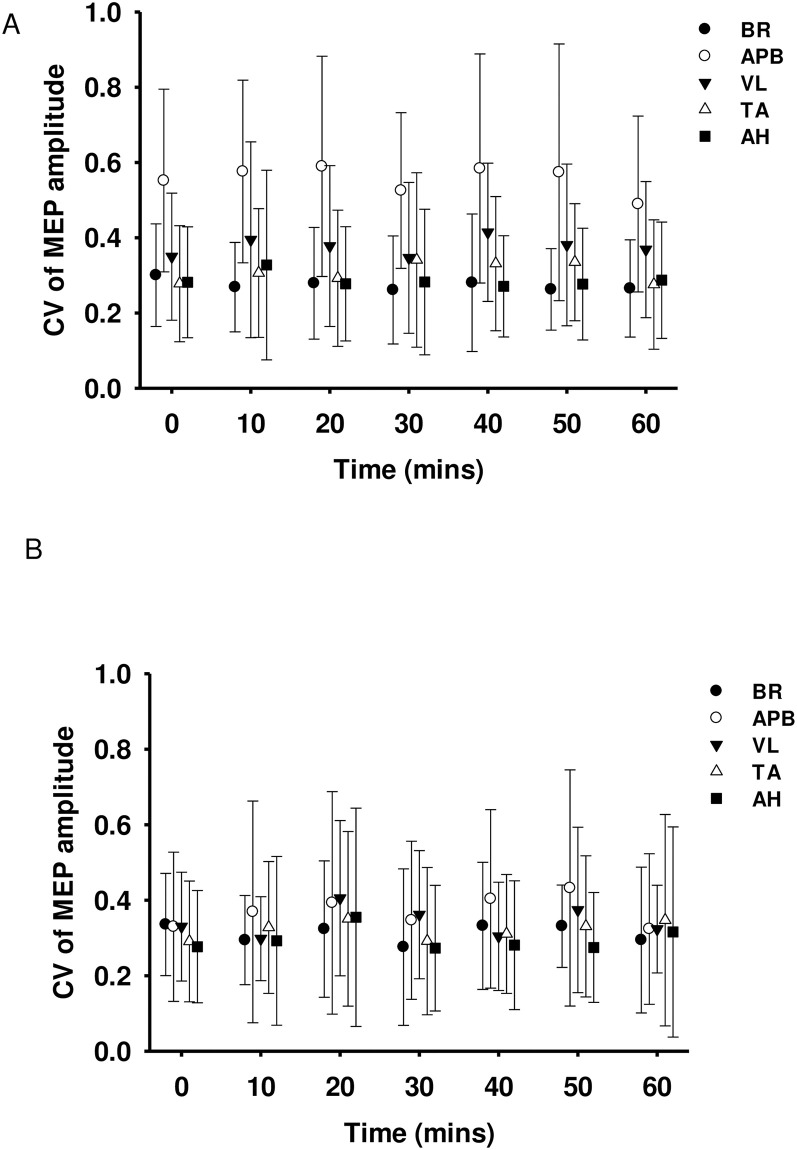
CV (mean ± SD) of MEP amplitude at each 10-minute time point for A) patients and B) healthy participants. BR = brachioradialis, APB = abductor pollicis brevis, VL = vastus lateralis, TA = tibialis anterior and AH = abductor hallucis.

There were no differences over time for any muscle in either the patients or the healthy participants (P>0.99 in all cases), see Fig 6.

### Correlations

#### VascuQol score and MEP amplitudes

There was no correlation between VascuQol score and MEP amplitudes (ρ range -0.37 to 0.07, P>0.05 in all cases) for any muscle, except VL (ρ -0.78, P<0.001). For VL, disease severity score was inversely proportional to MEP amplitude, i.e. worse patient-reported symptoms were associated with larger MEP amplitudes.

#### VascuQol score and MEP latency

There was no correlation between VascuQol score and MEP latency (ρ range -0.41 to -0.08 P>0.05 in all cases).

#### Age and avMEP amplitude

There was no correlation between age and avMEP amplitudes (ρ range- 0.31 to -0.10, P>0.05 in all cases) for any muscle except BR (ρ -0.55, P<0.001), where increases in age were associated with smaller avMEP amplitudes.

#### Age and avMEP latency

All muscles showed a significant positive correlation between increasing age and avMEP latency (ρ range 0.39 to 0.61, P<0.05 in all cases), i.e. latencies were longer in older participants.

#### avMEP amplitude and CV of avMEP amplitude

There were variable relationships between avMEP amplitudes and CVs of avMEP amplitudes. Significant relationships were found for APB and AH in healthy participants and for VL and TA in patients. The slopes of the significant correlations were always negative, i.e. increased avMEP amplitudes were associated with smaller CVs (APB healthy participants, ρ—0.47, P = 0.04; AH healthy participants ρ—0.54, P = 0.01; VL patients, ρ—0.52, P = 0.02; TA patients ρ—0.50, P = 0.02).

## Discussion

The results of this study have demonstrated MEP characteristics of patients with PVD. When compared to healthy, younger participants, they appear to be similar, with differences likely attributable to age rather than disease. In the following, these findings will be discussed further, along with their potential impact on future neurophysiological research and clinical application.

### MEP amplitude and latency

The data from this study showed no differences in the MEP amplitude and latency between PVD patients and healthy participants. When MEP amplitudes and latency were correlated with age for all participants, only BR showed a negative correlation with age, whilst all muscle latencies increased with increasing age. Although the healthy participants were not aged matched to PVD patients, this cohort was younger than PVD patients. Therefore, any differences observed appear to be due to the effects of aging, rather than due to pathology. In support of this, no differences in MEP amplitudes or latencies were seen between the symptomatic (presumably more diseased) and asymptomatic legs of PVD patients, allowing the data to be averaged.

Aging can have variable effects on MEP characteristics, with results in the literature being inconsistent. Some studies which compared healthy younger participants with matched healthy older participants, have found both at rest and during contraction, older participants have smaller MEP amplitudes with longer latencies [28, 29], the latter in keeping with the results of the current study. However, other studies have demonstrated that the effect of age on MEP amplitudes can vary between muscles [30–32]. One study, for example, showed input-output curves (stimulus intensity vs MEP amplitudes) of MEPs were different in an upper limb muscle, the first dorsal interosseous muscle, between younger and older adults but were the same for a lower limb muscle, vastus lateralis [31]. This is consistent with our data, where with increasing age, BR (an upper limb muscle) MEP amplitude appears to decrease, whilst no such difference was seen in the amplitude of lower limb muscle MEPs.

Further, disease severity scores showed no correlation between more severe disease and effects on MEP characteristics. A single significant correlation was found between the disease severity score and avMEP amplitude; higher VascuQol scores were associated with smaller VL amplitude. The VascuQol is a functional scoring system, assessing the patient’s ability to perform common daily activities, such as bathing; a maximum score of 7 would indicate ‘maximum’ function with no impact on function due to PVD. Therefore, with more severe disease (i.e. a lower VascuQol score), one would predict smaller MEPs secondary to potential tissue damage. No such correlations were found, and the single negative correlation is difficult to explain physiologically. It is therefore proposed symptomatic PVD, which impairs activities of daily living, appears to have little impact on limb muscle MEP characteristics.

The protocol was designed to include patients who had stable disease with symptomatic leg pain on exercise, not severe enough to require surgery; the mean VascuQol score reflects this. These patients have an arterial blood supply to their lower limbs which is adequate provided no exertional stress is placed upon them; there is a state of intermittent ischaemia. Based on our results, the severity of PVD in our patient cohort, and the severity of the ischaemia, does not seem to result in any deterioration of nerve function. The normal MEP characteristics is especially surprising given the muscle atrophy and sarcopenia that is observed many PVD patients, who find the claudication pain limits even simple daily tasks and physical activity [33]. It may be that patients with more severe PVD, such as those with critical limb ischaemia presenting with pain at rest and tissue necrosis, or sudden acute limb ischaemia following limb trauma for example, may have nerve dysfunction which manifests in a reduction in MEP amplitude or greatly prolonged latency. It has been shown experimentally that acute limb ischaemia following a tourniquet-induced ischaemic nerve block results in smaller MEPs with increased latencies [34, 35]. To the best of our knowledge however, the impact of moderate PVD on MEP characteristics has not been investigated before and provides valuable baseline data for older patients with known cardiovascular morbidity, with or without diagnosed PVD (given identical risk factors) undergoing neurophysiological investigation or surgery where IONM is employed.

We were aware of the potential effect of MEP amplitude on the variability of MEPs within a muscle. The design of our protocol meant there was a disparity between the relative stimulation intensity for each muscle given a single ‘combined’ test intensity per coil was used. A stimulus intensity of 120% of motor threshold of the muscle with the highest threshold was used; therefore, the intensity would be greater for the remaining muscles with lower motor thresholds. Previous work has shown greater stimulation intensities result in MEPs with not only larger amplitudes but also less variability, likely through consistent activation of most of the available motor units [25, 36, 37]. However, our results showed no consistent significant correlations between the mean CV of avMEP and the mean amplitude of avMEP for either group. However, significant correlations were always in the expected direction, with larger MEP amplitudes being associated with smaller CVs. Given the differences in strength of the circular coil and the cone coil, it is not surprising that the stimulus intensities used for the circular and cone coils were equivalent. However, the results also reveal that for a given coil, the intensities used for the two groups were not different, demonstrating that the thresholds for the patients were not higher.

Amplitude is a key factor to take into consideration when considering the use of MEPs for clinical purposes. A sudden reduction in amplitude or loss of MEPs is highly suggestive of injury during spinal surgery [38]. Muscles with small MEP amplitudes can make it difficult to detect injury-induced decreases. Furthermore, the depressive effects anaesthetic agents significantly reduce MEP amplitudes and prolong latencies [18, 39]. In many IONM guidelines, usually a hand and foot muscle are used as control and monitoring muscle, respectively [40]. The results of this study suggest (Fig 2B) that MEPs in APB (hand) and AH (foot) in PVD patients produced the largest mean amplitudes of the upper and lower limb muscles, respectively, similarly observed in healthy participants; thus they would likely be suitable for monitoring purposes in the PVD population as per standard IOMN guidelines [40].

### Reliability

Many neurophysiological studies have investigated the variability of TMS-induced MEPs [10, 27, 36, 41–43]. No previous study to our knowledge has compared the variability of different muscles over a prolonged time period. As can be seen from the current data, it is evident that there is variability in TMS-induced MEP characteristics between stimuli at each epoch and over time. Our interest lay in the extent of this variation over time within a given muscle.

During surgeries where the spinal cord is vulnerable, there will be certain surgical interventions or time points where there is a higher SCI risk. At these crucial timepoints, there will be a need for repeated trains of stimuli to continually assess spinal cord integrity, hence the need to assess variability within trains at a given time point (CV of mean MEP). Determining the variability of TMS-induced MEPs over time is also necessary particularly where frequent but intermittent monitoring is required for a longer duration of time, hence the need to assess variability over an extended period (CV of avMEP). This was achieved with a protocol recording trains of six MEPs at regular intervals over a 1-hour period.

TMS-induced MEPs have been shown to vary in amplitude [37]; this variability is greater with TMS than with TES, since motor cortical activation occurs largely trans-synaptically [27, 44]. TES induces MEPs through direct activation of corticospinal neurons and generates more consistent results [45]. It is for this reason that surgical neuromonitoring guidelines usually recommend TES [15]. However, there are practical disadvantages to its use. TES routinely utilises invasive needle or screw stimulating electrodes, compared to the non-invasive hand-held stimulating coil employed for TMS use. The use of high current stimulus intensities of TES is also very painful, usually restricting clinical use to the anaesthetised patient. This prevents interrogation of the motor pathways post-operatively in the lightly sedated or awake patient who may develop late SCI sometime after a TAAA repair; as many as 19% of patients develop paralysis 24 hours after this surgery, outside of the operating theatre, usually when spinal cord monitoring has ended [46].

Our results have shown that the CVs of the mean avMEP amplitude are comparable in patients and healthy participants. The mean CV of the avMEP amplitude gives an indication of the variation of all MEPs generated in each muscle over the course of the 1-hour protocol. This is especially true for BR and AH, which had the lowest CVs of the avMEP amplitude for upper and lower limb muscles in both groups of participants. These results are in keeping with previous studies, showing TMS-related measurements have good intra- and inter-investigator [41] and test-retest [43] reliabilities in healthy young and old participants [10, 37, 47]. Given there were no significant differences between healthy participants and PVD patient CV values in this study, the variability of normative MEP data in the literature where predominantly young, healthy participants are included, should be applicable to patients with cardiovascular disease or risk factors participating in research or neurophysiological testing.

Some of the variability in MEPs could be attributed to methodology [23, 48, 49], such as stimulus intensity or number of stimuli delivered, [10] level of muscle contraction at time of stimulation, [26, 50] data analysis procedures including removal of initial MEPs when averaging [36] and technical factors relating to coil position [51, 52]. To limit this, we delivered trains of 6 stimuli for our averaging calculations, maintained strict coil orientation and position, performed stimulation at rest (as confirmed by pre-stimulus EMG levels) and removed only data where there was muscle contraction. As a result, MEP measures in this investigation, including variability, are comparable to previously published work [27, 37, 53].

### Limitations

The authors acknowledge the comparison with data from younger healthy volunteers, rather than healthy age-matched participants, may be viewed as a limitation. The healthy volunteer data were generated from a protocol development study, undertaken before the current study. The comparison presented here is to demonstrate differences between diseased and disease-free participants. PVD is a disease of aging and associated with lifestyle factors, with the atherosclerotic process increasing with age; age is an independent risk factor, irrespective of health status [54]. Thus, it would be difficult to find an older person who would be truly disease-free and “healthy” with respect to their arterial wall integrity. Confirmation would require advanced functional assessments and blood flow imaging studies, [55] which would have been practically prohibitive for this study. Data exist for healthy older participants and our data are compared to the literature in the discussion.

The use of a cRMT results in a stimulation intensity of 120% for the muscle with the highest motor threshold, but greater for the other muscles. It is convention to use a specific coil and test intensity to target each muscle but this would have been impractical in the confines of a time critical protocol. However, previous work has shown only small differences in motor threshold between limb muscles, [3, 56] thus the use of a single test intensity is unlikely to have a major impact on the results obtained.

In many neurophysiological studies, MEP amplitudes are often normalised to the maximum M-waves (mMax), the maximal EMG response to direct motor nerve stimulation; [57] this allows comparisons to be made across muscles and between participants with different MEP amplitudes. However, since a key focus of the current investigation was variability of MEPs over time (as assessed by CV), normalisation to mMax was not deemed necessary as CV is a form of normalisation. Further, due to the time critical nature of this study design, it was not feasible to include measurement of MMax for all muscles studies.

### Conclusions & recommendations

MEPs were recordable from upper and lower limb muscles in PVD patients and were found to have similar characteristics to data from our healthy participants. Our results suggest there are no additional effects of pathological limb ischaemia on MEP characteristics beyond those expected by aging. With an increasingly aging population, there will be greater numbers of patients with PVD and cardiovascular risk factors participating in neurophysiological studies or undergoing surgery where spinal cord integrity is monitored [58] and MEPs from these patients can be easily evoked and interpreted.

## References

[1] Chiou SY, Hurry M, Reed T, Quek JX, Strutton PH. Cortical contributions to anticipatory postural adjustments in the trunk. J Physiol. 2018;596(7):1295–306. doi: 10.1113/JP275312 29368403 PMC5878228

[2] Chiou SY, Shih YF, Chou LW, McGregor AH, Strutton PH. Impaired neural drive in patients with low back pain. Eur J Pain. 2014;18(6):794–802. doi: 10.1002/j.1532-2149.2013.00428.x 24895331

[3] Nicotra A, King NK, Catley M, Mendoza N, McGregor AH, Strutton PH. Evaluation of corticospinal excitability in cervical myelopathy, before and after surgery, with transcranial magnetic stimulation: a pilot study. Eur Spine J. 2013;22(1):189–96. doi: 10.1007/s00586-012-2554-y 23132280 PMC3540307

[4] Cacchio A, Paoloni M, Cimini N, Mangone M, Liris G, Aloisi P, et al. Reliability of TMS-related measures of tibialis anterior muscle in patients with chronic stroke and healthy subjects. J Neurol Sci. 2011;303(1–2):90–4. doi: 10.1016/j.jns.2011.01.004 21262510

[5] Tanaka Y, Kawaguchi M, Noguchi Y, Yoshitani K, Kawamata M, Masui K, et al. Systematic review of motor evoked potentials monitoring during thoracic and thoracoabdominal aortic aneurysm open repair surgery: a diagnostic meta-analysis. J Anesth. 2016. doi: 10.1007/s00540-016-2242-x 27612851

[6] Yu T, Li QJ, Zhang XW, Wang Y, Jiang QY, Zhu XJ, et al. Multimodal intraoperative monitoring during surgical correction of scoliosis to avoid neurologic damage. Medicine (Baltimore). 2019;98(15):e15067. doi: 10.1097/MD.0000000000015067 30985657 PMC6485779

[7] Bisdas T, Panuccio G, Sugimoto M, Torsello G, Austermann M. Risk factors for spinal cord ischemia after endovascular repair of thoracoabdominal aortic aneurysms. J Vasc Surg. 2015;61(6):1408–16. doi: 10.1016/j.jvs.2015.01.044 25827967

[8] Stackelberg O, Wolk A, Eliasson K, Hellberg A, Bersztel A, Larsson SC, et al. Lifestyle and Risk of Screening-Detected Abdominal Aortic Aneurysm in Men. J Am Heart Assoc. 2017;6(5). doi: 10.1161/JAHA.116.004725 28490522 PMC5524061

[9] Behrendt CA, Thomalla G, Rimmele DL, Petersen EL, Twerenbold R, Debus ES, et al. Prevalence of peripheral arterial disease, abdominal aortic aneurysm, and risk factors in the Hamburg City Health Study: A Cross-Sectional Analysis. Eur J Vasc Endovasc Surg. 2023. 10.1016/j.ejvs.2023.01.002 36634745

[10] Christie A, Fling B, Crews RT, Mulwitz LA, Kamen G. Reliability of motor-evoked potentials in the ADM muscle of older adults. J Neurosci Methods. 2007;164(2):320–4. doi: 10.1016/j.jneumeth.2007.05.011 17588673

[11] Soda J, Pavelin S, Vujovic I, Rogic Vidakovic M. Assessment of Motor Evoked Potentials in Multiple Sclerosis. Sensors (Basel). 2023;23(1). 10.3390/s23010497 PMC9824873 36617096

[12] Dulfer SE, Lange F, Wapstra FH, Potgieser ARE, Valk JP, Absalom AR, et al. Intraoperative neurophysiological monitoring during scoliosis surgery in patients with Duchenne muscular dystrophy. Eur Spine J. 2020. doi: 10.1007/s00586-020-06458-9 32440770

[13] Glasby MA, Tsirikos AI, Henderson L, Horsburgh G, Jordan B, Michaelson C, et al. Transcranial magnetic stimulation in the semi-quantitative, pre-operative assessment of patients undergoing spinal deformity surgery. Eur Spine J. 2016. doi: 10.1007/s00586-016-4737-4 27554347

[14] Benjamin EJ, Blaha MJ, Chiuve SE, Cushman M, Das SR, Deo R, et al. Heart Disease and Stroke Statistics-2017 Update: A Report From the American Heart Association. Circulation. 2017;135(10):e146–e603. doi: 10.1161/CIR.0000000000000485 28122885 PMC5408160

[15] MacDonald DB. Overview on Criteria for MEP Monitoring. J Clin Neurophysiol. 2017;34(1):4–11. doi: 10.1097/WNP.0000000000000302 28045852

[16] Legatt AD. Ellen R. Grass Lecture: Motor evoked potential monitoring. Am J Electroneurodiagnostic Technol. 2004;44(4):223–43. 15675733

[17] Galloway GM, Dias BR, Brown JL, Henry CM, Brooks DA 2nd, Buggie EW. Transcranial magnetic stimulation—may be useful as a preoperative screen of motor tract function. J Clin Neurophysiol. 2013;30(4):386–9. doi: 10.1097/WNP.0b013e31829ddeb2 23912578

[18] Lee WY, Hou WY, Yang LH, Lin SM. Intraoperative monitoring of motor function by magnetic motor evoked potentials. Neurosurgery. 1995;36(3):493–500. doi: 10.1227/00006123-199503000-00008 7753349

[19] Rossi S, Antal A, Bestmann S, Bikson M, Brewer C, Brockmoller J, et al. Safety and recommendations for TMS use in healthy subjects and patient populations, with updates on training, ethical and regulatory issues: Expert Guidelines. Clin Neurophysiol. 2021;132(1):269–306. doi: 10.1016/j.clinph.2020.10.003 33243615 PMC9094636

[20] Morgan MB, Crayford T, Murrin B, Fraser SC. Developing the Vascular Quality of Life Questionnaire: a new disease-specific quality of life measure for use in lower limb ischemia. J Vasc Surg. 2001;33(4):679–87. doi: 10.1067/mva.2001.112326 11296317

[21] Met R, Reekers JA, Koelemay MJ, Legemate DA, de Haan RJ. The AMC linear disability score (ALDS): a cross-sectional study with a new generic instrument to measure disability applied to patients with peripheral arterial disease. Health Qual Life Outcomes. 2009;7:88. doi: 10.1186/1477-7525-7-88 19822016 PMC2766362

[22] Hermens HJ, Freriks B, Disselhorst-Klug C, Rau G. Development of recommendations for SEMG sensors and sensor placement procedures. J Electromyogr Kinesiol. 2000;10(5):361–74. doi: 10.1016/s1050-6411(00)00027-4 11018445

[23] Day BL, Dressler D, Maertens de Noordhout A, Marsden CD, Nakashima K, Rothwell JC, Thompson PD. Electric and magnetic stimulation of human motor cortex: surface EMG and single motor unit responses. J Physiol. 1989;412:449–73. doi: 10.1113/jphysiol.1989.sp017626 2489409 PMC1190586

[24] Rossini PM, Barker AT, Berardelli A, Caramia MD, Caruso G, Cracco RQ, et al. Non-invasive electrical and magnetic stimulation of the brain, spinal cord and roots: basic principles and procedures for routine clinical application. Report of an IFCN committee. Electroencephalogr Clin Neurophysiol. 1994;91(2):79–92. doi: 10.1016/0013-4694(94)90029-9 7519144

[25] Pellegrini M, Zoghi M, Jaberzadeh S. The effect of transcranial magnetic stimulation test intensity on the amplitude, variability and reliability of motor evoked potentials. Brain Res. 2018;1700:190–8. doi: 10.1016/j.brainres.2018.09.002 30194017

[26] Hess CW, Mills KR, Murray NM. Responses in small hand muscles from magnetic stimulation of the human brain. J Physiol. 1987;388:397–419. doi: 10.1113/jphysiol.1987.sp016621 3079553 PMC1192555

[27] Ellaway PH, Davey NJ, Maskill DW, Rawlinson SR, Lewis HS, Anissimova NP. Variability in the amplitude of skeletal muscle responses to magnetic stimulation of the motor cortex in man. Electroencephalogr Clin Neurophysiol. 1998;109(2):104–13. doi: 10.1016/s0924-980x(98)00007-1 9741800

[28] Tobimatsu S, Sun SJ, Fukui R, Kato M. Effects of sex, height and age on motor evoked potentials with magnetic stimulation. J Neurol. 1998;245(5):256–61. doi: 10.1007/s004150050215 9617705

[29] Matamala JM, Nunez C, Lera L, Verdugo RJ, Sanchez H, Albala C, et al. Motor evoked potentials by transcranial magnetic stimulation in healthy elderly people. Somatosens Mot Res. 2013;30(4):201–5. doi: 10.3109/08990220.2013.796922 23767989

[30] Nguyen DTA, Julkunen P, Saisanen L, Maatta S, Rissanen SM, Lintu N, et al. Developmental models of motor-evoked potential features by transcranial magnetic stimulation across age groups from childhood to adulthood. Sci Rep. 2023;13(1):10604. doi: 10.1038/s41598-023-37775-w 37391521 PMC10313665

[31] Rozand V, Senefeld JW, Sundberg CW, Smith AE, Hunter SK. Differential effects of aging and physical activity on corticospinal excitability of upper and lower limb muscles. J Neurophysiol. 2019;122(1):241–50. doi: 10.1152/jn.00077.2019 31091158 PMC6689774

[32] Pitcher JB, Ogston KM, Miles TS. Age and sex differences in human motor cortex input-output characteristics. J Physiol. 2003;546(Pt 2):605–13. doi: 10.1113/jphysiol.2002.029454 12527746 PMC2342521

[33] Ferreira JMM, Cunha P, Carneiro A, Vila I, Cunha C, Silva C, et al. Sarcopenia as a Prognostic Factor in Peripheral Arterial Disease: Descriptive Review. Ann Vasc Surg. 2021;74:460–74. doi: 10.1016/j.avsg.2021.01.076 33556522

[34] Werhahn KJ, Mortensen J, Kaelin-Lang A, Boroojerdi B, Cohen LG. Cortical excitability changes induced by deafferentation of the contralateral hemisphere. Brain. 2002;125(Pt 6):1402–13. doi: 10.1093/brain/awf140 12023328

[35] Brasil-Neto JP, Valls-Sole J, Pascual-Leone A, Cammarota A, Amassian VE, Cracco R, et al. Rapid modulation of human cortical motor outputs following ischaemic nerve block. Brain. 1993;116 (Pt 3):511–25. doi: 10.1093/brain/116.3.511 8513390

[36] Hashemirad F, Zoghi M, Fitzgerald PB, Jaberzadeh S. Reliability of Motor Evoked Potentials Induced by Transcranial Magnetic Stimulation: The Effects of Initial Motor Evoked Potentials Removal. Basic Clin Neurosci. 2017;8(1):43–50. doi: 10.15412/J.BCN.03080106 28446949 PMC5396172

[37] Kiers L, Cros D, Chiappa KH, Fang J. Variability of motor potentials evoked by transcranial magnetic stimulation. Electroencephalogr Clin Neurophysiol. 1993;89(6):415–23. doi: 10.1016/0168-5597(93)90115-6 7507428

[38] Macdonald DB, Skinner S, Shils J, Yingling C, American Society of Neurophysiological M. Intraoperative motor evoked potential monitoring—a position statement by the American Society of Neurophysiological Monitoring. Clin Neurophysiol. 2013;124(12):2291–316. doi: 10.1016/j.clinph.2013.07.025 24055297

[39] Legatt AD, Emerson Rg Fau—Epstein CM, Epstein Cm Fau—MacDonald DB, MacDonald Db Fau—Deletis V, Deletis V Fau—Bravo RJ, Bravo Rj Fau—Lopez JR, et al. ACNS Guideline: Transcranial Electrical Stimulation Motor Evoked Potential Monitoring. 2016(1537–1603 (Electronic)). 10.1097/WNP.0000000000000253 26756258

[40] Grover H, Walsh P, Sanders B, Shirley C. UPDATED ANS/BSCN GUIDELINES FOR NEUROPHYSIOLOGICAL RECORDINGS OF THE SPINAL CORD DURING CORRECTIVE SPINAL DEFORMITY SURGERY: British Society for Clinical Neurophysiology; 2018 [https://www.bscn.org.uk/data/files/Guidelines/IOM_guide2.pdf.

[41] Cacchio A, Cimini N, Alosi P, Santilli V, Marrelli A. Reliability of transcranial magnetic stimulation-related measurements of tibialis anterior muscle in healthy subjects. Clin Neurophysiol. 2009;120(2):414–9. doi: 10.1016/j.clinph.2008.11.019 19135412

[42] Hassanzahraee M, Zoghi M, Jaberzadeh S. Longer TMS inter-trial interval increases size, reduces variability and improves reliability of the motor evoked potentials. Brain Connect. 2019. 10.1089/brain.2019.0714 31744309

[43] Hermsen AM, Haag A, Duddek C, Balkenhol K, Bugiel H, Bauer S, et al. Test-retest reliability of single and paired pulse transcranial magnetic stimulation parameters in healthy subjects. J Neurol Sci. 2016;362:209–16. doi: 10.1016/j.jns.2016.01.039 26944150

[44] Truccolo WA, Ding M, Knuth KH, Nakamura R, Bressler SL. Trial-to-trial variability of cortical evoked responses: implications for the analysis of functional connectivity. Clin Neurophysiol. 2002;113(2):206–26. doi: 10.1016/s1388-2457(01)00739-8 11856626

[45] Day BL, Thompson PD, Dick JP, Nakashima K, Marsden CD. Different sites of action of electrical and magnetic stimulation of the human brain. Neurosci Lett. 1987;75(1):101–6. doi: 10.1016/0304-3940(87)90083-8 3574763

[46] Etz DC, Luehr M, Aspern KV, Misfeld M, Gudehus S, Ender J, et al. Spinal cord ischemia in open and endovascular thoracoabdominal aortic aneurysm repair: new concepts. J Cardiovasc Surg (Torino). 2014;55(2 Suppl 1):159–68. 24796909

[47] Sivaramakrishnan A, Madhavan S. Stimulus Intensity Affects Variability of Motor Evoked Responses of the Non-Paretic, but Not Paretic Tibialis Anterior Muscle in Stroke. Brain Sci. 2020;10(5). doi: 10.3390/brainsci10050297 32429115 PMC7287783

[48] Rothwell JC, Thompson PD, Day BL, Boyd S, Marsden CD. Stimulation of the human motor cortex through the scalp. Exp Physiol. 1991;76(2):159–200. doi: 10.1113/expphysiol.1991.sp003485 2059424

[49] Rothwell JC. Techniques and mechanisms of action of transcranial stimulation of the human motor cortex. J Neurosci Methods. 1997;74(2):113–22. doi: 10.1016/s0165-0270(97)02242-5 9219881

[50] Darling WG, Wolf SL, Butler AJ. Variability of motor potentials evoked by transcranial magnetic stimulation depends on muscle activation. Experimental brain research Experimentelle Hirnforschung Experimentation cerebrale. 2006;174(2):376–85. doi: 10.1007/s00221-006-0468-9 16636787 PMC3582032

[51] Barker AT, Jalinous R, Freeston IL. Non-invasive magnetic stimulation of human motor cortex. Lancet. 1985;1(8437):1106–7. doi: 10.1016/s0140-6736(85)92413-4 2860322

[52] Chipchase L, Schabrun S, Cohen L, Hodges P, Ridding M, Rothwell J, et al. A checklist for assessing the methodological quality of studies using transcranial magnetic stimulation to study the motor system: an international consensus study. Clin Neurophysiol. 2012;123(9):1698–704. doi: 10.1016/j.clinph.2012.05.003 22647458 PMC4884647

[53] Burke D, Hicks R, Stephen J, Woodforth I, Crawford M. Trial-to-trial variability of corticospinal volleys in human subjects. Electroencephalogr Clin Neurophysiol. 1995;97(5):231–7. doi: 10.1016/0013-4694(95)00005-j 7489684

[54] Thijssen DH, Carter SE, Green DJ. Arterial structure and function in vascular ageing: are you as old as your arteries? (1469–7793 (Electronic)). 10.1113/JP270597 PMC4933112 26140618

[55] Hirsch AT, Haskal ZJ, Hertzer NR, Bakal CW, Creager MA, Halperin JL, et al. ACC/AHA 2005 guidelines for the management of patients with peripheral arterial disease (lower extremity, renal, mesenteric, and abdominal aortic): executive summary a collaborative report from the American Association for Vascular Surgery/Society for Vascular Surgery, Society for Cardiovascular Angiography and Interventions, Society for Vascular Medicine and Biology, Society of Interventional Radiology, and the ACC/AHA Task Force on Practice Guidelines (Writing Committee to Develop Guidelines for the Management of Patients With Peripheral Arterial Disease) endorsed by the American Association of Cardiovascular and Pulmonary Rehabilitation; National Heart, Lung, and Blood Institute; Society for Vascular Nursing; TransAtlantic Inter-Society Consensus; and Vascular Disease Foundation. J Am Coll Cardiol. 2006;47(6):1239–312. doi: 10.1016/j.jacc.2005.10.009 16545667

[56] Strutton PH, Catley M, McGregor AH, Davey NJ. Corticospinal excitability in patients with unilateral sciatica. Neurosci Lett. 2003;353(1):33–6. doi: 10.1016/j.neulet.2003.09.005 14642431

[57] Clos P, Mater A, Legrand H, Poirier G, Ballay Y, Martin A, et al. Corticospinal Excitability Is Lower During Eccentric Than Concentric Cycling in Men. Front Physiol. 2022;13:854824. doi: 10.3389/fphys.2022.854824 35370788 PMC8966379

[58] Lin HS, Watts JN, Peel NM, Hubbard RE. Frailty and post-operative outcomes in older surgical patients: a systematic review. BMC Geriatr. 2016;16(1):157. doi: 10.1186/s12877-016-0329-8 27580947 PMC5007853

